# Supplementary material for: Circulating Multiple Myeloma Cells (CMMCs) as Prognostic and Predictive Markers in Multiple Myeloma and Smouldering MM Patients
Source: Cancers (Basel). 2024 Aug 23;16(17):2929. doi: 10.3390/cancers16172929 (PMC11393854; doi:10.3390/cancers16172929)
Supplement: Supplementary file 1 [file cancers-16-02929-s001.zip › cancers-3124078-supplementary.pdf]

**Table S1.** Clinical information. MM cohort's characteristics are listed.

| **Induction therapy** | **Patients n=(%)** |
|---|---|
| Non-antiCD38-based | 23 (52) |
| AntiCD38-based | 21 (48) |
| **Induction response** | |
| ≥VGPR | 24 (60) |
| <VGPR | 16 (40) |
| **Transplant (TX)** | |
| Yes | 40 (91) |
| No | 4 (9) |
| **TX response** | |
| ≥VGPR | 21 (54) |
| <VGPR | 18 (46) |

VGPR=very good partial response; TX=transplant

**Table S2.** CMMCs count, single cell separation, and genomic analysis quality results.

| **Samples** | **CMMCs count** | **CMMCs redetected in DEPArray (%)** | **CMMCs recovered (%)** | **CMMCs passing Ampli1 WGA QC (%)** | **CMMCs passing Ampli1 LowPass QC (%)** | **CMMCs with interpretable CNA profiles (%)** | **CMMCs Aberrant (%)** |
|---|---|---|---|---|---|---|---|
| CMMC01 | 15 | 5 (33%) | 5 (100%) | 3 (60%) | 3 (100%) | 3 (100%) | 3 (100%) |
| CMMC02 | 16 | 7 (44%) | 2 (29%) | 2 (100%) | 2 (100%) | 2 (100%) | 2 (100%) |
| CMMC03 | 32 | 13 (41%) | 10 (77%) | 8 (89%) | 6 (75%) | 6 (100%) | 6 (100%) |
| CMMC04 | 62 | 42 (68%) | 23 (55%) | 21 (95%) | 21 (100%) | 21 (100%) | 19 (90%) |
| TOTAL | 125 | 67 (54%) | 40 (60%) | 34 (85%) | 32 (94%) | 32 (100%) | 30 (94%) |

WGA=whole genome amplification; QC=quality control; CNA=copy number alteration

**Table S3.** Biochemical parameters at different timepoint for each patient, with the relative CMMCs count.

| ***Patient ID*** | **Pathology** | **Time-point (m)** | **CMMCs count** | **Total protein (g/dL)** | **M-protein (%)** | **M-protein (g/dL)** | **LDH (IU/L)** | **Creatinine (mg/dL)** | **β2G (%)** | **B2G (g/dL)** | **K/L ratio** | **Calcium (mg/dL)** | **CRP (mg/dL)** | **Albumin (%)** | **Albumin (g/dL)** | **WBC (x10^9/L)** | **Hemoglobin (g/dL)** | **Hematocrit (%)** | **Platelets (x10^9/L)** | **β2M (mg/L)** |
|---|---|---|---|---|---|---|---|---|---|---|---|---|---|---|---|---|---|---|---|---|
| *AIRC19_001* | SMM | 17 | 22 | 8.4 | 31.5 | 2.65 | 117.1 | 1.02 | 40.2 | 3.38 | 0.04 | 9.40 | 0.04 | 40.7 | 3.42 | 3.65 | 13.60 | 39.90 | 260 | 3.60 |
| *AIRC19_001* | SMM | 21 | 2 | 8.7 | 31.6 | 2.75 | 120.6 | 1.15 | 40.6 | 3.53 | 0.03 | 9.27 | 0.19 | 39.8 | 3.46 | 4.01 | 13.60 | 42.20 | 267 | 4.79 |
| *AIRC19_001* | SMM | 38 | 52 | 8.6 | 32.7 | 2.81 | 124.9 | 1.10 | 40.9 | 3.52 | 0.04 | 9.35 | 0.04 | 40.8 | 3.51 | 4.52 | 14.40 | 44.60 | 259 | 3.47 |
| *AIRC19_004* | MM | 0 | | | | | 160 | 1.15 | | | 24.40 | 9.9 | 0.1 | | 4.40 | | 10.40 | | 234 | 3.4 |
| *AIRC19_004* | MM | 9 | 0 | | | | | | | | | | | | | | | | | |
| *AIRC19_004* | MM | 12 | 2 | 6.42 | 4.5 | 0.29 | 171.3 | 1.08 | 3.6 | 0.23 | 3.48 | 9.14 | 0.23 | 64.1 | 4.12 | 3.56 | 9.60 | 27.70 | 144 | |
| *AIRC19_012* | MM | 0 | | | | | 163 | 0.65 | | | | 9.3 | 0.27 | | | | 11.60 | | 237 | |
| *AIRC19_012* | MM | 18 | 0 | | | | | | | | | | | | | 4.65 | 11.70 | 35.90 | 215 | |
| *AIRC19_013* | MM | 0 | 0 | 5.9 | 4.3 | 0.25 | 187.3 | 0.58 | 4.9 | 0.29 | 10.79 | 8.70 | 0.28 | 57.2 | 3.37 | 3.06 | 12.20 | 35.00 | | 2.74 |
| *AIRC19_013* | SMM | 12 | 24 | 7.2 | 16.2 | 1.17 | 162.5 | 0.60 | 4.7 | 0.34 | 65.42 | 8.80 | 0.23 | 50.0 | 3.60 | 4.84 | 11.80 | 36.60 | 298 | |
| *AIRC19_013* | SMM | 23 | 100 | 7.0 | 14.9 | 1.04 | 157.0 | 0.60 | 4.5 | 0.32 | 67.19 | 8.83 | 0.20 | 50.9 | 3.56 | 5.62 | 12.40 | 38.00 | 303 | 2.51 |

| | | | | | | | | | | | | | | | | | | | | |
|---|---|---|---|---|---|---|---|---|---|---|---|---|---|---|---|---|---|---|---|---|
| *AIRC19_013* | MM | 30 | 4 | 7.1 | 17.9 | 1.27 | 161.6 | 0.67 | 4.5 | 0.32 | 105.86 | 8.72 | 0.20 | 50.3 | 3.57 | 4.79 | 12.30 | 37.50 | 247 | 2.50 |
| *AIRC19_014* | MM | 0 | 371 | | | | 156 | 0.87 | | | | 9.50 | 0.46 | | | | | | 436.0 | 1.90 |
| *AIRC19_014* | MM | 7 | 1 | 6.6 | 10.6 | 0.70 | 140.9 | 0.67 | 3.1 | 0.20 | | 9.60 | 0.38 | 64.4 | 4.24 | 22.59 | 12.00 | 34.00 | | 1.80 |
| *AIRC19_014* | MM | 12 | 8 | 7.4 | 10.3 | 0.76 | 146.8 | 0.86 | 3.0 | 0.22 | 2.85 | 10.04 | 0.07 | 63.2 | 4.68 | 4.94 | 12.80 | 37.10 | 202.0 | 1.81 |
| *AIRC19_016* | MM | 0 | | | | 2.00 | 137 | 1.97 | | | 16.00 | 11.5 | 1.38 | | 3.28 | | 13.50 | | 264 | 6.1 |
| *AIRC19_016* | MM | 5 | 0 | | | | | | | | | | | | | | | | | |
| *AIRC19_016* | MM | 10 | 1 | 5.2 | x | x | 139 | 1.86 | 4.3 | 0.22 | 1.41 | 8.7 | 2.85 | 67.8 | 3.53 | 3.16 | 8.30 | 22.10 | 16 | 3.49 |
| *AIRC19_018* | MM | 0 | | | | | 243 | 0.89 | | | | 11.4 | 0.07 | | 4.12 | | 9.00 | | 435 | 5.7 |
| *AIRC19_018* | MM | 4 | 0 | | | | | | | | | | | | | | | | | |
| *AIRC19_018* | MM | 7 | 0 | 6.39 | 1.1 | 0.07 | 139.3 | 1.23 | 3.2 | 0.20 | 2.83 | 9.5 | 0.07 | 71.5 | 4.57 | 33.26 | 8.80 | 25.30 | 67 | |
| *AIRC19_019* | MM | 0 | | | | | 167 | 0.75 | | | | 9.2 | 0.23 | | 4.30 | | 14.80 | | 232 | |
| *AIRC19_019* | MM | 3 | 0 | | | | | | | | | | | | | | | | | |
| *AIRC19_019* | MM | 6 | 3 | 6.8 | 1.3 | 0.09 | 227 | 0.87 | 4.6 | 0.31 | 3.05 | 10 | 0.25 | 66.8 | 4.54 | 4.49 | 13.60 | 41.30 | 237 | |
| *AIRC19_019* | MM | 8 | 12 | 6.6 | 1.3 | 0.09 | 263 | 0.67 | 4.5 | 0.30 | 3.17 | 9.5 | 2.6 | 66.3 | 4.38 | 8.98 | 11.20 | 31.90 | 38 | |
| *AIRC19_020* | MM | 0 | | | | | 234 | 0.64 | | | 489.00 | 9.3 | 0.24 | | 4.00 | | 12.00 | | 174 | 1.6 |
| *AIRC19_020* | MM | 3 | 62 | | | | | | | | | | | | | | | | | |
| *AIRC19_020* | MM | 8 | 0 | 5.2 | x | x | 199 | 0.68 | 5.4 | 0.28 | 0.94 | 8.7 | 0.8 | 59.7 | 3.10 | 0.14 | 10.10 | 27.60 | 42 | 2 |
| *AIRC19_021* | SMM | 2 | 52 | | | | | | | | | | | | | | | | | |
| *AIRC19_021* | SMM | 7 | 159 | 10.0 | 31.3 | 3.13 | 162.4 | 0.81 | 40.2 | 4.02 | 0.03 | 11.30 | 0.67 | 43.1 | 4.31 | 5.85 | 12.20 | 39.00 | 316 | 1.91 |
| *AIRC19_021* | SMM | 13 | 49 | 9.2 | 37.3 | 3.43 | 160.0 | 0.85 | 41.7 | 3.84 | 0.03 | 11.04 | 0.55 | 43.0 | 3.96 | 5.38 | 11.60 | 37.00 | 247 | 2.26 |
| *AIRC19_021* | SMM | 26 | 138 | 10.5 | 35.7 | 3.75 | 160.1 | 0.75 | 42.4 | 4.45 | 0.03 | 11.93 | 0.46 | 41.0 | 4.31 | 5.84 | 12.40 | 39.90 | 241 | 1.99 |
| *AIRC19_023* | MM | 0 | 55 | | | | 155 | 5.39 | | | | 10.07 | | | | | | | 586.0 | 5.00 |
| *AIRC19_024* | MM | 0 | | 12.0 | | 49.00 | 150 | 0.67 | 50.0 | 6.00 | | 9.70 | 0.21 | 34.0 | 4.08 | | 12.60 | | 262.0 | 2.60 |
| *AIRC19_024* | MM | 1 | 16 | 5.6 | x | x | 194.5 | 0.78 | 3.8 | 0.21 | 0.00 | 9.12 | 0.84 | 62.3 | 3.49 | 4.95 | 11.70 | 34.40 | 236 | |
| *AIRC19_024* | MM | 5 | 1 | 5.8 | 0.9 | 0.05 | 179.9 | 0.59 | 4.5 | 0.26 | 0.01 | 9.12 | 0.22 | 65.7 | 3.81 | 3.69 | 12.50 | 36.00 | 200 | |
| *AIRC19_024* | MM | 5 | 0 | 4.96 | x | x | 104.1 | 0.66 | 5 | 0.25 | | 8.08 | 3.47 | 61.5 | 3.05 | 0.22 | 11.10 | 31.60 | 69 | |
| *AIRC19_024* | MM | 12 | 0 | 5.75 | x | x | 183.7 | 0.74 | 4.1 | 0.24 | 0.90 | 9.57 | 0.17 | 66.1 | 3.80 | 2.33 | 12.20 | 35.30 | 140 | |
| *AIRC19_026* | MM | 0 | 133 | | | | 140 | 0.71 | | 0.00 | | 9.10 | 3.50 | | 0.00 | | | | 222.0 | 3.29 |
| *AIRC19_026* | MM | 3 | 5 | 6.6 | 6.7 | 0.44 | 206.9 | 0.75 | 4.5 | 0.30 | 2.04 | 8.89 | 0.67 | 57.0 | 3.76 | 4.03 | 11.60 | 33.90 | 314.0 | |
| *AIRC19_026* | MM | 11 | 5 | 6.0 | x | x | 263.8 | 0.56 | 5.0 | 0.30 | 1.00 | 9.03 | | | 0.00 | 5.45 | 10.60 | 33.20 | 292.0 | 3.83 |
| *AIRC19_027* | MM | 0 | 18588 | | | | 171 | 1.52 | | 0.00 | | 8.00 | 3.22 | | 0.00 | | | | 154.0 | 49.70 |
| *AIRC19_027* | MM | 3 | 15 | 6.1 | x | x | 227.1 | 0.99 | | 0.00 | | 9.15 | | | 0.00 | 2.34 | 11.90 | 35.20 | 233.0 | |
| *AIRC19_027* | MM | 11 | 0 | 5.7 | 6.7 | 0.38 | 192.9 | 0.93 | 3.3 | 0.19 | 3.13 | 9.27 | 0.11 | 63.8 | 3.64 | 3.16 | 12.40 | 36.20 | 99.0 | 3.91 |
| *AIRC19_027* | MM | 18 | 3 | 5.5 | 6.0 | 0.33 | 208.9 | 1.01 | 4.2 | 0.23 | 0.74 | 9.14 | 1.23 | 67.3 | 3.67 | 4.59 | 11.70 | 33.70 | 120.0 | |
| *AIRC19_028* | MM | 0 | | 8.4 | 27.4 | 2.30 | 143 | 0.96 | 2.2 | 0.18 | 4.38 | 9.10 | 0.03 | 49.4 | 4.15 | 8.67 | 10.60 | 33.70 | 202.0 | 3.90 |
| *AIRC19_028* | MM | 1 | 54 | 9.6 | 32.8 | 3.15 | 135 | 1.01 | 1.8 | 0.17 | 7.73 | 9.3 | 0.04 | 47.4 | 4.55 | 7.13 | 10.60 | 31.20 | 142 | 3.8 |
| *AIRC19_028* | MM | 17 | 2 | 6.5 | 2.9 | 0.19 | 161 | 0.84 | 3.5 | 0.23 | | 9.5 | 0.02 | 69.2 | 4.50 | 5.45 | 11.70 | 33.80 | 143 | 1.5 |
| *AIRC19_029* | MM | 0 | 1 | 8.2 | 18.8 | 1.54 | 184.9 | 0.71 | 2.6 | 0.21 | 319.50 | 10.23 | 0.03 | 57.6 | 4.71 | 5.68 | 12.50 | 37.50 | 326.0 | 1.52 |
| *AIRC19_029* | MM | 8 | 0 | 6.5 | 6.8 | 0.44 | 228.4 | 0.67 | 4.1 | 0.27 | 21.53 | 9.78 | 0.02 | 63.6 | 4.13 | 10.42 | 11.20 | 33.80 | 382.0 | 1.40 |
| *AIRC19_030* | MM | 1 | 316 | 7.4 | 9.9 | 0.73 | 183.8 | 0.6 | 4.0 | 0.30 | 0.01 | 9.42 | 0.11 | 60.8 | 4.50 | 6.55 | 11.90 | 36.90 | 344.0 | 1.20 |
| *AIRC19_030* | MM | 6 | 0 | 6.0 | x | | 133 | 0.55 | 3.7 | 0.22 | 0.63 | 8.86 | 0.13 | 67.6 | 4.06 | 5.18 | 12.40 | 38.10 | 279.0 | 1.38 |
| *AIRC19_030* | MM | 10 | 6 | 6.0 | 1.0 | 0.06 | 160.7 | 0.58 | 3.7 | 0.22 | 0.80 | 9.38 | 0.08 | 68.2 | 4.09 | 4.09 | 11.30 | 37.80 | 255.0 | 1.71 |
| *AIRC19_030* | MM | 13 | 0 | 5.8 | 1.1 | 0.06 | 166.8 | 0.56 | 3.6 | 0.21 | 0.58 | 9.07 | 0.12 | 69.4 | 4.03 | 2.85 | 11.10 | 33.50 | 219.0 | |
| *AIRC19_031* | MM | 0 | 15 | 7.4 | 18.8 | 1.39 | 424 | 0.76 | 2.9 | 0.21 | 7.03 | 9.3 | 0.22 | 49.3 | 3.65 | 5.01 | 10.50 | 29.80 | 227 | |
| *AIRC19_031* | MM | 0 | | 10.8 | 32.3 | 3.49 | 814 | 0.89 | 1.9 | 0.21 | 41.53 | 9.90 | 0.09 | 40.6 | 4.38 | 5.85 | 10.90 | 32.60 | 186.0 | 4.40 |
| *AIRC19_031* | MM | 6 | 0 | 7.1 | 6.8 | 0.48 | 299 | 0.72 | 3.3 | 0.23 | 2.05 | 10 | 0.12 | 61.6 | 4.37 | 5.60 | 11.20 | 33.20 | 181 | 2.9 |

| | | | | | | | | | | | | | | | | | | | | |
|---|---|---|---|---|---|---|---|---|---|---|---|---|---|---|---|---|---|---|---|---|
| *AIRC19_031* | MM | 10 | 0 | 6.4 | 2.9 | 0.19 | 195 | 0.79 | 3.6 | 0.23 | 2.00 | 9.7 | 0.06 | 64.7 | 4.14 | 5.93 | 11.40 | 33.30 | 170 | |
| *AIRC19_033* | MM | 0 | | 8 | 21.9 | 1.75 | 275 | 0.73 | 3.4 | 0.27 | 142.07 | 9 | 0.78 | 49.7 | 3.98 | 4.51 | 18.80 | 37.70 | 206 | 2.01 |
| *AIRC19_033* | MM | 1 | 65 | 8.3 | 24.1 | 2.00 | 226 | 0.73 | 3.5 | 0.29 | 97.43 | 8.9 | 0.34 | 50 | 4.15 | 5.46 | 12.60 | 37.30 | 220 | 2.1 |
| *AIRC19_033* | MM | 6 | 897 | 6.5 | 1.7 | 0.11 | 178 | 0.62 | 5.4 | 0.35 | 1.34 | 9.2 | 0.33 | 58.7 | 3.82 | 3.74 | 10.20 | 32.60 | 188 | |
| *AIRC19_033* | MM | 11 | 0 | 6.7 | x | x | 209 | 0.88 | 3.8 | 0.25 | 1.34 | 9.3 | 0.36 | 61.8 | 4.14 | 4.35 | 10.90 | 34.40 | 227 | |
| *AIRC19_035* | MM | 0 | 39940 | 9.0 | 48.4 | 4.36 | 109.4 | 0.93 | 2.6 | 0.23 | 47.18 | 7.57 | 4.45 | 23.8 | 2.14 | 7.18 | 8.90 | 26.30 | 146.0 | 8.37 |
| *AIRC19_035* | MM | 6 | 0 | 6.7 | 5.7 | 0.38 | 150 | 0.86 | 3.3 | 0.22 | 15.22 | 9.03 | 0.59 | 62.8 | 4.21 | 6.06 | 13.60 | 38.00 | 116.0 | 3.21 |
| *AIRC19_036* | MM | 0 | 736 | 6.8 | 1.3 | 0.09 | 173 | 1.43 | 12.6 | 0.86 | 0.03 | 10.04 | 0.05 | 66.0 | 4.49 | 5.45 | 11.00 | 32.70 | 175.0 | 4.18 |
| *AIRC19_036* | MM | 4 | 0 | 6.4 | 1.5 | 0.10 | 179.8 | 0.68 | 3.6 | 0.23 | 0.70 | 9.58 | 0.10 | 70.4 | 4.51 | 5.79 | 15.00 | 44.80 | 171.0 | |
| *AIRC19_036* | MM | 15 | 0 | 6.2 | 1.5 | 0.09 | 189 | 0.73 | 4.4 | 0.27 | 1.51 | 9.80 | 0.20 | 69.3 | 4.30 | 6.11 | 14.10 | 41.70 | 222.0 | |
| *AIRC19_037* | MM | 0 | 576 | 10.6 | 36.7 | 3.89 | 155.3 | 0.88 | 55.0 | 5.83 | 197.52 | 8.68 | 0.07 | 32.4 | 3.43 | 2.16 | 10.10 | 30.50 | 181.0 | 4.41 |
| *AIRC19_037* | MM | 4 | 18 | 8.1 | 34.2 | 2.78 | 195.3 | 1.04 | 43.6 | 3.55 | 191.55 | 8.67 | 0.14 | 40.5 | 3.30 | 2.27 | 11.10 | 34.20 | 178.0 | |
| *AIRC19_037* | MM | 8 | 0 | 6.1 | 8.3 | 0.51 | 307 | 1.1 | 17.6 | 1.07 | 21.27 | 8.74 | 0.31 | 59.1 | 3.61 | 2.79 | 10.00 | 28.90 | 223.0 | 3.00 |
| *AIRC19_037* | MM | 14 | 1 | 6.0 | 1.2 | 0.07 | 281.3 | 1 | 3.6 | 0.22 | 1.41 | 9.95 | 0.19 | 71.8 | 4.31 | 3.54 | 12.00 | 35.80 | 164.0 | 4.72 |
| *AIRC19_038* | MM | 0 | 2335 | 9.1 | 24.1 | 2.19 | 175.5 | 0.57 | 3.7 | 0.34 | 1.82 | 9.27 | 3.62 | 38.3 | 3.49 | 6.12 | 12.10 | 37.80 | 294.0 | 2.97 |
| *AIRC19_038* | MM | 7 | 0 | 6.2 | x | x | 253.9 | 0.68 | 4.5 | 0.28 | 1.93 | 9.67 | 0.90 | 68.3 | 4.23 | 5.12 | 11.70 | 35.20 | 170.0 | 2.16 |
| *AIRC19_038* | MM | 17 | 0 | 5.8 | x | x | 258.3 | 0.51 | 5.3 | 0.31 | 0.82 | 7.59 | 9.29 | 56.6 | 3.28 | 4.57 | 10.20 | 31.20 | 141.0 | |
| *AIRC19_039* | MM | 0 | | 9.7 | 41.8 | 4.05 | 109 | 1.19 | 50.5 | 4.90 | 0.02 | 9.10 | 0.18 | 31.9 | 3.09 | 3.41 | 9.70 | 29.10 | 129.0 | 3.86 |
| *AIRC19_039* | MM | 1 | 9 | 7.6 | 2.5 | 0.19 | 145 | 0.81 | 29.8 | 2.26 | 0.12 | 9 | 0.22 | 46.8 | 3.56 | 4.36 | 12.60 | 39.20 | 295 | |
| *AIRC19_039* | MM | 2 | 0 | 6 | 4.5 | 0.27 | 203 | 0.71 | 12.3 | 0.74 | 0.71 | 8.9 | 0.28 | 61.3 | 3.68 | 4.16 | 13.70 | 41.20 | 346 | |
| *AIRC19_039* | MM | 6 | 5 | 6.4 | 3.9 | 0.25 | 216 | 0.93 | 9.9 | 0.63 | 1.01 | 9.1 | 0.47 | 59.8 | 3.83 | 5.26 | 15.90 | 46.70 | 236 | |
| *AIRC19_039* | MM | 12 | 2 | 6.9 | 2.3 | 0.16 | 202 | 0.99 | 8 | 0.55 | 0.75 | 9.8 | 0.33 | 62.4 | 4.31 | 5.82 | 17.30 | 48.80 | 185 | |
| *AIRC19_040* | MM | 0 | 1491 | 5.5 | 0.5 | 0.03 | 355.5 | 0.7 | 5.1 | 0.28 | 514.73 | 8.56 | 0.75 | 54.7 | 3.01 | 7.29 | 8.50 | 26.20 | 213.0 | 10.27 |
| *AIRC19_051* | MM | 0 | 960 | 7.0 | 14.3 | 1.00 | 192.1 | 0.78 | 3.2 | 0.22 | 67.29 | 8.97 | 0.15 | 57.9 | 4.05 | 3.98 | 14.00 | 42.60 | | 2.47 |
| *AIRC19_051* | SMM | 1 | 976 | 6.7 | 15.9 | 1.07 | 188.2 | 0.81 | 3.3 | 0.22 | 29.42 | 9.09 | 0.16 | 57.1 | 3.83 | 5.16 | 13.80 | 42.60 | 280 | 2.26 |
| *AIRC19_053* | MM | 0 | | 7.9 | 10.9 | 0.86 | 205.6 | 0.56 | 19.2 | 1.52 | 1.86 | 9.86 | 0.15 | 51.0 | 4.03 | 4.55 | 11.50 | 34.60 | 444 | 1.97 |
| *AIRC19_053* | MM | 1 | 27 | 6.4 | 2.8 | 0.18 | 225 | 0.6 | 8.4 | 0.54 | 1.24 | 8.9 | 1.17 | 55.4 | 3.55 | 5.29 | 11.90 | 35.90 | 251 | |
| *AIRC19_053* | MM | 5 | 21 | 5.9 | x | x | 326 | 0.58 | 4.2 | 0.25 | 0.94 | 9.3 | 0.43 | 62.1 | 3.66 | 45.08 | 11.70 | 35.60 | 243 | |
| *AIRC19_053* | MM | 15 | 79 | 6.2 | x | x | 278 | 0.51 | 4.5 | 0.28 | 0.94 | 7.8 | 0.26 | 57.3 | 3.55 | 5.23 | 10.80 | 32.80 | 354 | 1.7 |
| *AIRC19_054* | MM | 0 | 38 | 10.0 | 36.0 | 3.60 | 107.0 | 0.92 | 52.1 | 5.21 | 0.03 | 9.03 | 0.17 | 34.7 | 3.47 | 4.48 | 13.10 | 38.00 | | 3.04 |
| *AIRC19_054* | SMM | 1 | 327 | 9.7 | 35.8 | 3.47 | 94.2 | 0.95 | 50.0 | 4.85 | 0.03 | 9.39 | 0.06 | 36.6 | 3.55 | 5.02 | 13.90 | 40.60 | 159 | |
| *AIRC19_054* | MM | 4 | 10 | 6.5 | 1.4 | 0.09 | 180.6 | 0.73 | 7.1 | 0.46 | 1.17 | 9.30 | 0.04 | 65.2 | 4.24 | 6.31 | 13.80 | 40.60 | | |
| *AIRC19_054* | MM | 9 | 28 | 7.2 | 2.9 | 0.21 | 182.4 | 1.01 | 9.2 | 0.66 | 1.01 | 9.78 | 0.07 | 62.1 | 4.47 | 8.16 | 15.40 | 44.60 | 167 | 1.68 |
| *AIRC19_054* | MM | 13 | 19 | 7.7 | 2.6 | 0.20 | 178.0 | 0.74 | 3.8 | 0.29 | 1.72 | 9.43 | 0.06 | 60.6 | 4.67 | 6.12 | 13.10 | 37.80 | 131 | 2.51 |
| *AIRC19_055* | MM | 3 | 0 | 5.5 | 1.0 | 0.06 | 199.8 | 1.09 | 4.7 | 0.26 | 0.55 | 8.52 | 5.19 | 56.0 | 3.08 | 6.72 | 10.40 | 31.70 | 258 | |
| *AIRC19_055* | SMM | 4 | 2463 | 7.8 | 15.0 | 1.17 | 161.0 | 0.99 | 3.1 | 0.24 | 0.08 | 9.26 | 0.34 | 52.6 | 4.10 | 6.29 | 10.60 | 34.20 | 159 | 3.33 |
| *AIRC19_055* | MM | 7 | 0 | 5.8 | | | 211.2 | 1.06 | 4.4 | 0.26 | 0.93 | 8.93 | 1.63 | 58.6 | 3.40 | 5.42 | 11.20 | 34.70 | 224 | 3.59 |
| *AIRC19_055* | SMM | 11 | 390 | 8.0 | 14.3 | 1.14 | 161.0 | 1.19 | 3.5 | 0.28 | 0.06 | 9.10 | 0.55 | 48.5 | 3.88 | 4.93 | 9.90 | 32.40 | 143 | |
| *AIRC19_055* | MM | 16 | 619 | 7.6 | 16.3 | 1.24 | 185.5 | 1.35 | 3.8 | 0.29 | 0.07 | 9.79 | 0.48 | 48.2 | 3.66 | 5.76 | 9.60 | 29.00 | 188 | 3.91 |
| *AIRC19_056* | MM | 0 | | 6.9 | x | x | 165.4 | 0.56 | 5.2 | 0.36 | 389.02 | 9.45 | 0.06 | 60.9 | 4.19 | 7.22 | 13.90 | 39.70 | 322 | 2.31 |
| *AIRC19_056* | MM | 4 | 1 | 6.58 | x | x | 168.8 | 0.52 | 3.6 | 0.24 | 2.15 | 9.52 | 0.03 | 64.3 | 4.23 | 6.08 | 14.90 | 43.50 | 408 | 1.73 |
| *AIRC19_057* | MM | 0 | 13 | 9.1 | 19.1 | 1.75 | 186.7 | 0.77 | 3.1 | 0.28 | 19.58 | 9.39 | 0.19 | 44.4 | 4.06 | 5.07 | 12.50 | 37.00 | 312.0 | 2.08 |
| *AIRC19_057* | MM | 5 | 0 | 8.2 | 16.9 | 1.39 | 179.9 | 0.79 | 3.2 | 0.26 | 12.24 | 9.14 | 0.10 | 51.7 | 4.24 | 6.44 | 13.00 | 38.20 | 282.0 | |
| *AIRC19_057* | MM | 9 | 0 | 6.2 | 4.9 | 0.30 | 241.6 | 0.84 | 4.1 | 0.25 | 2.46 | 8.31 | 0.36 | 61.2 | 3.79 | 6.40 | 9.60 | 29.00 | 408.0 | 1.70 |
| *AIRC19_057* | MM | 15 | 12 | 7.2 | 3.1 | 0.22 | 193.1 | 0.99 | 4.2 | 0.30 | 2.35 | 9.24 | 0.35 | 60.4 | 4.35 | 5.17 | 10.90 | 32.40 | 255.0 | 2.14 |
| *AIRC19_058* | MM | 0 | | 10.7 | 36.0 | 3.85 | 143 | 0.87 | 3.5 | 0.37 | 75.33 | 10.00 | 1.52 | 35.1 | 3.76 | 7.25 | 13.00 | 38.50 | 354.0 | 3.50 |

| | | | | | | | | | | | | | | | | | | | | | |
|---|---|---|---|---|---|---|---|---|---|---|---|---|---|---|---|---|---|---|---|---|---|
| *AIRC19_058* | MM | 1 | 0 | 8.4 | 28.6 | 2.40 | 191 | 0.79 | 3.9 | 0.33 | 30.02 | 8.5 | 2.27 | 38.6 | 3.24 | 4.93 | 12.40 | 38.30 | 558 | |
| *AIRC19_058* | MM | 6 | 4 | 6.5 | 8.2 | 0.53 | 190 | 0.76 | 4.2 | 0.27 | 6.96 | 8.4 | 0.68 | 54.9 | 3.57 | 6.34 | 13.80 | 39.70 | 280 | |
| *AIRC19_058* | MM | 11 | 43 | 6.4 | 8.4 | 0.54 | 214 | 0.79 | 4.3 | 0.28 | 5.96 | 9 | 0.42 | 58.8 | 3.76 | 5.30 | 14.00 | 39.70 | 249 | 1.8 |
| *AIRC19_058* | MM | 15 | 7 | | | | | | | | | | | | | | | | | |
| *AIRC19_058* | MM | 19 | 180 | 8.5 | 28.4 | 2.41 | 155 | 0.77 | 3.7 | 0.31 | 643.25 | 9.1 | 8.66 | 38 | 3.23 | 8.16 | 11.00 | 32.50 | 362 | 21.8 |
| *AIRC19_059* | MM | 0 | | 11 | 32.5 | 3.58 | 185 | 0.93 | 2.2 | 0.24 | 40.93 | 8.9 | 0.28 | 38.9 | 4.28 | 5.29 | 8.80 | 26.90 | 190 | 5.15 |
| *AIRC19_059* | MM | 2 | 220 | 10.2 | 33.2 | 3.39 | 200 | 1.1 | 2.6 | 0.27 | 71.19 | 8 | 0.86 | 39.3 | 4.01 | 3.84 | 8.90 | 27.30 | 208 | |
| *AIRC19_059* | MM | 10 | 0 | 6.3 | 2.1 | 0.13 | 160 | 0.95 | 4 | 0.25 | 1.70 | 9 | 0.22 | 65.4 | 4.12 | 6.20 | 13.50 | 41.70 | 170 | |
| *AIRC19_059* | MM | 14 | 0 | 5.9 | 0.8 | 0.05 | 150 | 1.12 | 4.1 | 0.24 | 2.27 | 9.2 | 0.14 | 67.3 | 3.97 | 4.51 | 13.40 | 39.50 | 224 | |
| *AIRC19_059* | MM | 19 | 0 | 5.9 | 1.1 | 0.06 | 167 | 0.96 | 3.8 | 0.22 | 2.41 | 9.2 | 0.12 | 66.7 | 3.94 | 5.75 | 14.20 | 40.80 | 264 | |
| *AIRC19_060* | MM | 0 | 11 | 8.3 | 17.0 | 1.41 | 184 | 0.75 | 29.2 | 2.42 | 25.24 | 9.66 | 0.17 | 47.4 | 3.94 | 3.16 | 12.90 | 37.70 | 139.0 | 1.33 |
| *AIRC19_060* | MM | 4 | 2 | 6.2 | x | x | 271.5 | 0.71 | 3.3 | 0.20 | 1.67 | 9.36 | 0.36 | 67.3 | 4.17 | 4.92 | 13.20 | 41.10 | 194.0 | |
| *AIRC19_060* | MM | 9 | 1 | 6.5 | x | x | 283.2 | 0.8 | 3.2 | 0.21 | 1.07 | 9.48 | 0.16 | 69.5 | 4.52 | 2.75 | 14.30 | 40.70 | 161.0 | 1.40 |
| *AIRC19_060* | MM | 13 | 0 | 6.2 | x | x | 283.6 | 0.91 | 2.6 | 0.16 | 1.69 | 9.15 | 0.21 | 71.0 | 4.40 | 3.14 | 12.10 | 33.90 | 110.0 | 1.45 |
| *AIRC19_071* | MM | 0 | | 6.7 | 1.6 | 0.11 | 196.9 | 0.81 | 6.3 | 0.42 | 2.90 | 9.59 | 0.42 | 60.1 | 4.03 | 7.96 | 15.00 | 44.60 | 228.0 | 1.16 |
| *AIRC19_071* | MM | 1 | 0 | 6.52 | x | x | 162.1 | 0.85 | 6.7 | 0.44 | 1.29 | 9.6 | 0.95 | 60.5 | 3.94 | 5.13 | 15.10 | 44.60 | 319 | |
| *AIRC19_072* | MM | 0 | | 8.9 | 29.9 | 2.66 | 101 | 0.79 | 38.8 | 3.45 | 17.50 | 10.11 | 0.13 | 39.2 | 3.49 | 4.22 | 11.30 | 34.20 | 220 | 3.1 |
| *AIRC19_072* | MM | 1 | 1 | 7.9 | 21 | 1.66 | 120 | 0.87 | 31 | 2.45 | 9.86 | 9.5 | 0.79 | 42.8 | 3.38 | 2.82 | 11.50 | 35.10 | 368 | |
| *AIRC19_072* | MM | 5 | 12 | 7.6 | 21.2 | 1.61 | 121 | 0.73 | 36.7 | 2.79 | 16.53 | 8.9 | 0.28 | 41 | 3.12 | 3.75 | 11.60 | 34.90 | 351 | |
| *AIRC19_074* | MM | 0 | 0 | 8.4 | 24.8 | 2.08 | 140 | 0.94 | 2.8 | 0.24 | 0.15 | 9.8 | 0.19 | 46.2 | 3.88 | 3.88 | 13.30 | 37.30 | 207 | 2.8 |
| *AIRC19_074* | MM | 0 | | 8.01 | 22.8 | 1.83 | 143.2 | 1.01 | 2.6 | 0.21 | 0.16 | 9.56 | 0.24 | 46.8 | 3.75 | 4.08 | 12.40 | 35.90 | 243 | 2.78 |
| *AIRC19_074* | MM | 5 | 0 | 6.5 | 2 | 0.13 | 176 | 0.93 | 4.4 | 0.29 | 1.18 | 9.6 | 0.13 | 71.1 | 4.62 | 3.79 | 13.40 | 36.50 | 170 | |
| *AIRC19_074* | MM | 11 | 0 | 6 | x | x | 251 | 0.73 | 4.9 | 0.29 | 7.95 | 9.5 | 0.16 | 67.2 | 4.03 | 4.38 | 11.80 | 33.30 | 197 | |
| *AIRC19_075* | SMM | 8 | 344 | 6.5 | x | x | 196.1 | 0.78 | 4.5 | 0.29 | 96.69 | 10.13 | 0.30 | 67.5 | 4.39 | 4.69 | 14.80 | 42.60 | 163 | 1.57 |
| *AIRC19_075* | SMM | 12 | 656 | 6.3 | x | x | 209.7 | 0.93 | 4.0 | 0.25 | 97.68 | 9.69 | 0.16 | 71.2 | 4.49 | 4.84 | 15.60 | 46.10 | 145 | 1.72 |
| *AIRC19_075* | SMM | 16 | 371 | 6.2 | x | x | 215.3 | 0.90 | 4.0 | 0.25 | 125.25 | 9.52 | 0.17 | 70.2 | 4.35 | 4.99 | 15.30 | 45.70 | 131 | |
| *AIRC19_081* | MM | 0 | | 7.0 | 1.3 | 0.09 | 320.4 | 1.31 | 4.5 | 0.32 | 32.35 | 9.78 | 1.01 | 59.6 | 4.17 | 4.40 | 9.00 | 26.70 | 201 | 8.39 |
| *AIRC19_081* | MM | 3 | 0 | 5.54 | x | x | 128.9 | 0.8 | 4 | 0.22 | 1.27 | 8.82 | 0.59 | 68.5 | 3.79 | 2.64 | 11.10 | 34.30 | 218 | |
| *AIRC19_081* | MM | 9 | 0 | 5.94 | x | x | 184.6 | 0.99 | 4.4 | 0.26 | 0.85 | 9.07 | 0.2 | 66.6 | 3.96 | 5.17 | 11.80 | 35.40 | 307 | |
| *AIRC19_082* | MM | 0 | | 8.64 | 25.2 | 2.18 | 218.3 | 0.58 | 4.2 | 0.36 | 14.34 | 9.2 | 0.31 | 42.9 | 3.71 | 5.18 | 12.30 | 37.70 | 208 | 3.01 |
| *AIRC19_082* | MM | 2 | 0 | 5.7 | 2.8 | 0.16 | 161 | 0.82 | 3.9 | 0.22 | 1.55 | 9.1 | 0.20 | 66.3 | 3.78 | 4.93 | 11.80 | 36.10 | 298 | |
| *AIRC19_082* | MM | 10 | 0 | 6.8 | x | x | 216 | 0.88 | 4.1 | 0.28 | 1.83 | 8.9 | 0.11 | 35.3 | 2.40 | 4.11 | 12.40 | 38.10 | 84 | 1.7 |
| *AIRC19_083* | MM | 0 | | 9.7 | 32.3 | 3.13 | 164 | 0.66 | 2.2 | 0.21 | 41.79 | 8.9 | 0.06 | 39.1 | 3.79 | 3.64 | 11.20 | 31.90 | 279 | 2.6 |
| *AIRC19_083* | MM | 4 | 0 | 6 | 4.2 | 0.25 | 194 | 0.68 | 4 | 0.24 | 2.74 | 9.5 | 0.09 | 64.1 | 3.85 | 3.24 | 12.50 | 36.40 | 214 | |
| *AIRC19_083* | MM | 11 | 0 | 5.8 | 3.1 | 0.18 | 194 | 0.69 | 3.3 | 0.19 | 2.32 | 8.6 | 0.05 | 69.1 | 4.01 | 2.54 | 10.80 | 32.10 | 117 | |
| *AIRC19_084* | MM | 0 | | 11.6 | 52.1 | 6.04 | 85.5 | 0.74 | 61.2 | 7.10 | 257.20 | 10.37 | 0.03 | 28.1 | 3.26 | 6.10 | 9.40 | 28.60 | 130 | 3.19 |
| *AIRC19_084* | MM | 2 | 5432 | 10.82 | 43.2 | 4.67 | 95 | 0.71 | 1.9 | 0.21 | 317.92 | 10.14 | 0.11 | 30.4 | 3.29 | 2.70 | 11.50 | 35.30 | 232 | |
| *AIRC19_085* | MM | 0 | | 9.48 | 38.9 | 3.69 | 121 | 0.9 | 2 | 0.19 | 0.02 | 9.34 | 0.27 | 41.5 | 3.93 | 5.26 | 11.60 | 33.40 | 237 | 4 |
| *AIRC19_085* | MM | 5 | 333 | 6.1 | 6.5 | 0.40 | 155 | 0.77 | 3 | 0.18 | 0.33 | 9.4 | 0.13 | 68.3 | 4.17 | 4.11 | 12.40 | 36.70 | 279 | |
| *AIRC19_085* | MM | 10 | 68 | 6.4 | 7.9 | 0.51 | 153 | 0.87 | 3.2 | 0.20 | 0.16 | 9.3 | 0.16 | 65.7 | 4.20 | 3.68 | 12.70 | 37.30 | 195 | 2.19 |
| *AIRC19_088* | MM | 0 | | 9.3 | 37.2 | 3.46 | 132.6 | 1.14 | 2.8 | 0.26 | 0.06 | 8.92 | 0.22 | 40.4 | 3.76 | 6.41 | 13.70 | 38.00 | 246 | 2.4 |
| *AIRC19_088* | MM | 3 | 178 | 7.9 | 24.6 | 1.94 | 152 | 0.91 | 3.6 | 0.28 | 0.16 | 8.9 | 0.44 | 48.9 | 3.86 | 6.18 | 13.10 | 38.10 | 280 | |
| *AIRC19_088* | MM | 8 | 74 | 7.6 | 17.5 | 1.33 | 154 | 0.97 | 3.2 | 0.24 | 0.21 | 8.8 | 0.2 | 50.9 | 3.87 | 4.41 | 12.40 | 35.80 | 197 | 3.2 |
| *AIRC19_090* | MM | 0 | 171 | 7 | 34.7 | 2.43 | 123 | 0.8 | 4.6 | 0.32 | 69.00 | 7.9 | 1.19 | 34.5 | 2.42 | 4.79 | 12.30 | 33.90 | 171 | |
| *AIRC19_090* | MM | 0 | | 9.6 | 37.5 | 3.60 | 118.6 | 0.83 | 4.3 | 0.41 | 34.53 | 8.91 | 0.93 | 34.5 | 3.31 | 5.90 | 12.50 | 35.80 | 237 | 2.54 |
| *AIRC19_090* | MM | 5 | 0 | 6.5 | 7.9 | 0.51 | 190 | 0.67 | 5.3 | 0.34 | 2.60 | 9 | 0.68 | 61.2 | 3.98 | 5.51 | 12.00 | 37.20 | 352 | |

| | | | | | | | | | | | | | | | | | | | | |
|---|---|---|---|---|---|---|---|---|---|---|---|---|---|---|---|---|---|---|---|---|
| *AIRC19_091* | MM | 0 | | 8.46 | 20.5 | 1.73 | 218.1 | 0.76 | 4.1 | 0.35 | 0.03 | 10.15 | 2.63 | 41.5 | 3.51 | 10.39 | 14.50 | 42.30 | 215 | 1.79 |
| *AIRC19_091* | MM | 2 | 55 | 6.5 | 13.9 | 0.90 | 218 | 0.66 | 3.7 | 0.24 | 0.11 | 9.4 | 0.31 | 55.9 | 3.63 | 5.55 | 12.20 | 36.90 | 306 | |
| *AIRC19_091* | MM | 5 | 95 | 6.9 | 6.1 | 0.42 | 186 | 0.63 | 3.8 | 0.26 | 0.20 | 9.9 | 0.34 | 62.4 | 4.31 | 0.36 | 13.70 | 38.80 | 31 | |
| *AIRC19_091* | MM | 10 | 3 | 6.6 | 5.7 | 0.38 | 212 | 0.64 | 3.8 | 0.25 | 0.83 | 9.6 | 0.1 | 64.4 | 4.25 | 3.83 | 12.90 | 37.50 | 218 | |
| *AIRC19_093* | MM | 0 | | 6.87 | 1.3 | 0.09 | 208.9 | 0.92 | 3.3 | 0.23 | 0.00 | 9.86 | 0.36 | 68.6 | 4.71 | 5.76 | 14.30 | 41.80 | 130 | 3.12 |
| *AIRC19_093* | MM | 1 | 19 | 6.2 | 2 | 0.12 | 174 | 1.04 | 3.5 | 0.22 | | 9.9 | 0.58 | 69.2 | 4.29 | 1.82 | 13.70 | 41.10 | 101 | |
| *AIRC19_093* | MM | 5 | 0 | 5.2 | 1.6 | 0.08 | 199 | 0.78 | 4.4 | 0.23 | 0.02 | 9.4 | 1.98 | 65.9 | 3.43 | 2.53 | 13.10 | 37.60 | 79 | 2.3 |
| *AIRC19_093* | MM | 10 | 1 | 7.3 | x | x | 230 | 0.95 | 3.6 | 0.26 | 0.17 | 9.9 | 0.20 | 67.8 | 4.95 | 7.71 | 13.20 | 37.20 | 114 | |

m=months; LDH=lactate dehydrogenase; β2G=beta-2 globulin; CRP=C-reactive protein; WBC=white blood cell; β2M=beta-2 microglobulin; x=positive not-quantifiable (>0 and <0.01)

**Table S4.** Reference ranges accounted for in our laboratory for biochemical parameters.

| | Range Male | Range Female |
|---|---|---|
| **Total Protein (g/dL)** | 6.6-8.3 | 6.6-8.3 |
| **Serum M-Protein (%)** | absent | absent |
| **Serum M-Protein (g/dL)** | absent | absent |
| **Lactate Dehydrogenase (IU/L)** | <248 | <248 |
| **Serum Creatinine (mg/dL)** | 0.6-1.3 | 0.6-1.3 |
| **Beta-2 Globulin (%)** | 11.1-18.8 | 11.1-18.8 |
| **Beta-2 Globulin (g/dL)** | 0.7-1.5 | 0.7-1.5 |
| **Kappa/Lambda Ratio** | 0.26-1.65 | 0.26-1.65 |
| **Calcium (mg/dL)** | 8.6-10.5 | 8.6-10.5 |
| **C-Reactive Protein (mg/dL)** | <0.5 | <0.5 |
| **Serum Albumin (%)** | 55.8-66.1 | 55.8-66.1 |
| **Serum Albumin (g/dL)** | 3.3-5.7 | 3.3-5.7 |
| **White Blood Cells (x10^9/L)** | 3.6-10.5 | 3.6-10.5 |
| **Hemoglobin (g/dL)** | 13.5-17.2 | 12-15.6 |
| **Hematocrit (%)** | 39.5-50.5 | 35.5-45.5 |
| **Platelets (x10^9/L)** | 160-370 | 160-370 |
| **Serum Beta-2 Microglobulin (mg/L)** | 0.7-1.8 | 0.7-1.8 |
| **Creatinine Clearance (ml/min)** | 97-137 | 88-128 |

**Table S5**. BM NGS-based MRD measurements at different time-point for each patient and the relative CMMCs count.

| Patient ID | Time-point post treatment (m) | Results | Quantifications | CMMCs count |
|---|---|---|---|---|
| **AIRC19_082** | 3 | NEG | 0 | 0 |
| **AIRC19_081** | 4 | NEG | 0 | 0 |
| **AIRC19_083** | 4 | POS | 2.17E-04 | 0 |
| **AIRC19_038** | 6 | NEG | 0 | 0 |
| **AIRC19_029** | 6 | POS | 3.35E-03 | 0 |
| **AIRC19_082** | 10 | NEG | 0 | 0 |
| **AIRC19_083** | 10 | POS | 2.52E-04 | 0 |
| **AIRC19_004** | 10 | POS | 4.24E-04 | 0 |
| **AIRC19_018** | 11 | NEG | 0 | 0 |
| **AIRC19_033** | 11 | NEG | 0 | 0 |
| **AIRC19_029** | 11 | POS | 9.41E-03 | 0 |
| **AIRC19_020** | 12 | NEG | 0 | 0 |
| **AIRC19_083** | 13 | POS | 1.01E-05 | 0 |
| **AIRC19_012** | 14 | POS | 1.45E-03 | 0 |
| **AIRC19_060** | 15 | NEG | 0 | 0 |
| **AIRC19_036** | 16 | NEG | 0 | 0 |

| | | | | |
|---|---|---|---|---|
| **AIRC19_035** | 16 | POS | 1.00E-05 | 0 |
| **AIRC19_035** | 19 | POS | 4.24E-05 | 0 |
| **AIRC19_056** | 6 | POS | 1.00E-05 | 1 |
| **AIRC19_014** | 7 | POS | 3.22E+00 | 1 |
| **AIRC19_016** | 8 | NEG | 0 | 1 |
| **AIRC19_060** | 8 | NEG | 0 | 1 |
| **AIRC19_028** | 11 | NEG | 0 | 1 |
| **AIRC19_028** | 18 | POS | 9.22E-05 | 1 |
| **AIRC19_026** | 24 | NEG | 0 | 2 |
| **AIRC19_019** | 7 | POS | 8.25E-05 | 3 |
| **AIRC19_091** | 12 | POS | 1.29E-02 | 3 |
| **AIRC19_039** | 11 | POS | 2.19E-03 | 4 |
| **AIRC19_026** | 9 | NEG | 0 | 5 |
| **AIRC19_026** | 11 | NEG | 0 | 5 |
| **AIRC19_030** | 11 | NEG | 0 | 6 |
| **AIRC19_024** | 6 | POS | 1.06E-03 | 8 |
| **AIRC19_014** | 10 | POS | 2.70E-03 | 8 |
| **AIRC19_019** | 10 | POS | 2.83E-05 | 12 |
| **AIRC19_057** | 13 | NEG | 0 | 12 |
| **AIRC19_054** | 19 | NEG | 0 | 19 |
| **AIRC19_054** | 9 | POS | 2.06E-03 | 28 |
| **AIRC19_020** | 7 | POS | 2.91E-03 | 31 |
| **AIRC19_088** | 10 | POS | 3.86E-03 | 39 |
| **AIRC19_085** | 10 | POS | 2.85E-03 | 68 |
| **AIRC19_091** | 6 | POS | 5.38E-03 | 95 |
| **AIRC19_055** | 16 | POS | 1.00E-05 | 619 |

m=months; POS=detectable MRD; NEG=undetectable MRD

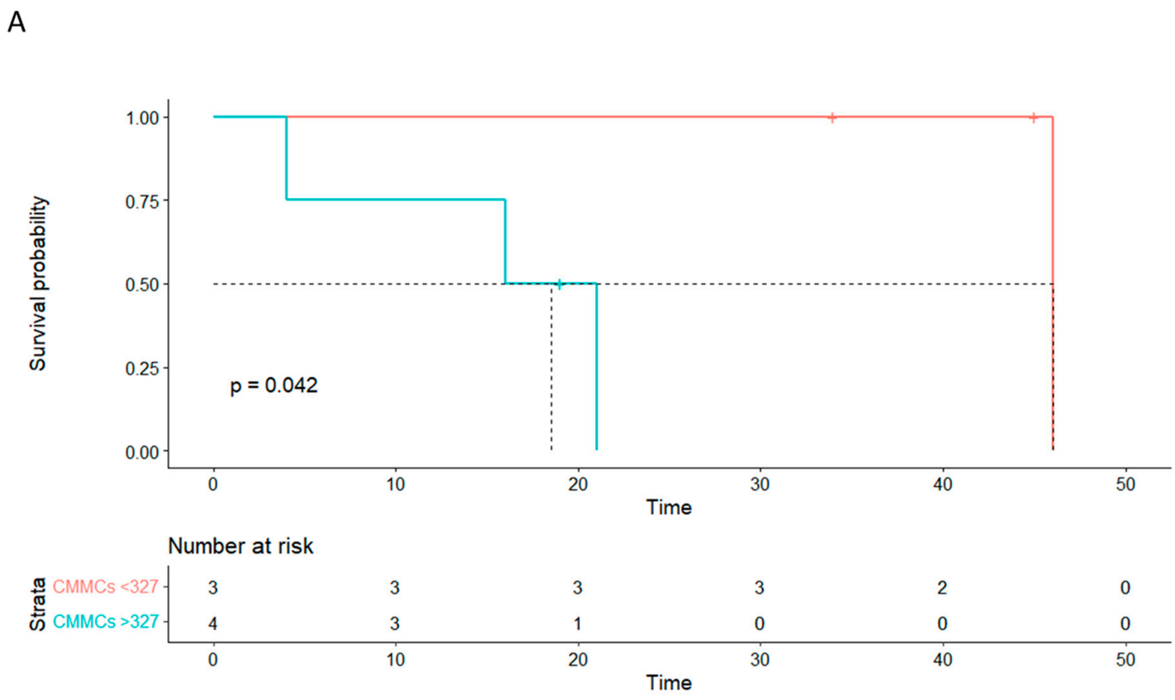


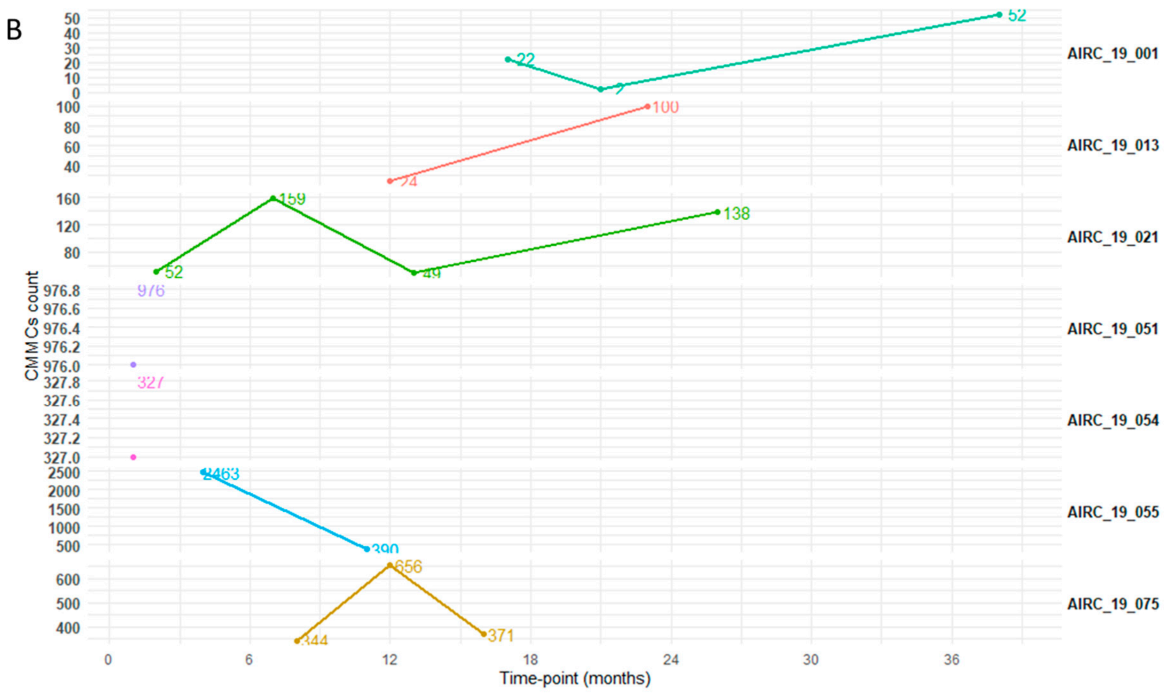


**Figure S1.** A) Time-to-progression to active MM according to CMMCs median basal levels (p=0.042). B) SMM line plot CMMCs kinetics highlighted *per* patient.

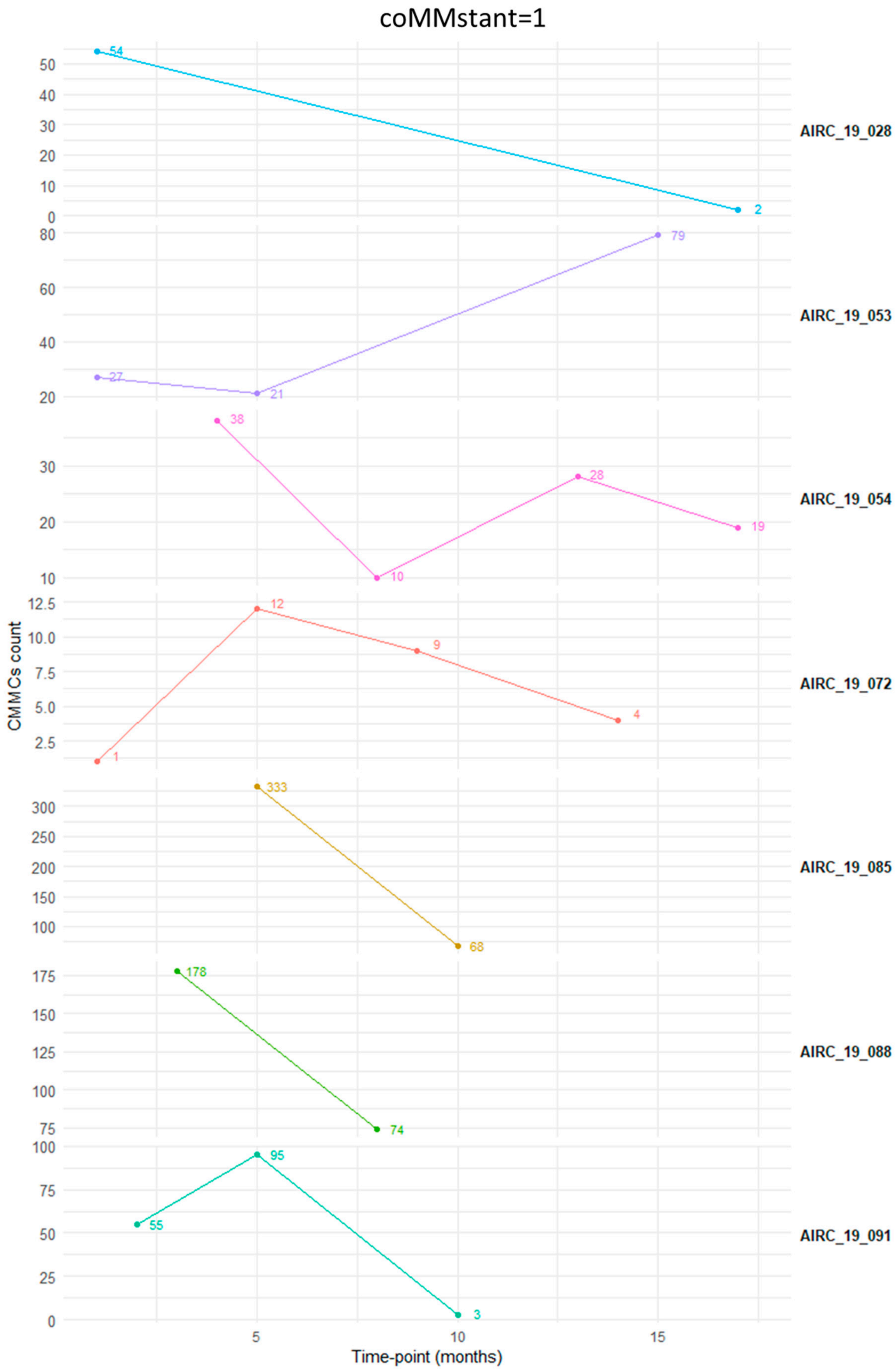


**Figure S2.** Line plot of CMMCs kinetics over time (in months) highlighted *per* patient in the context of coMMstant index=1.

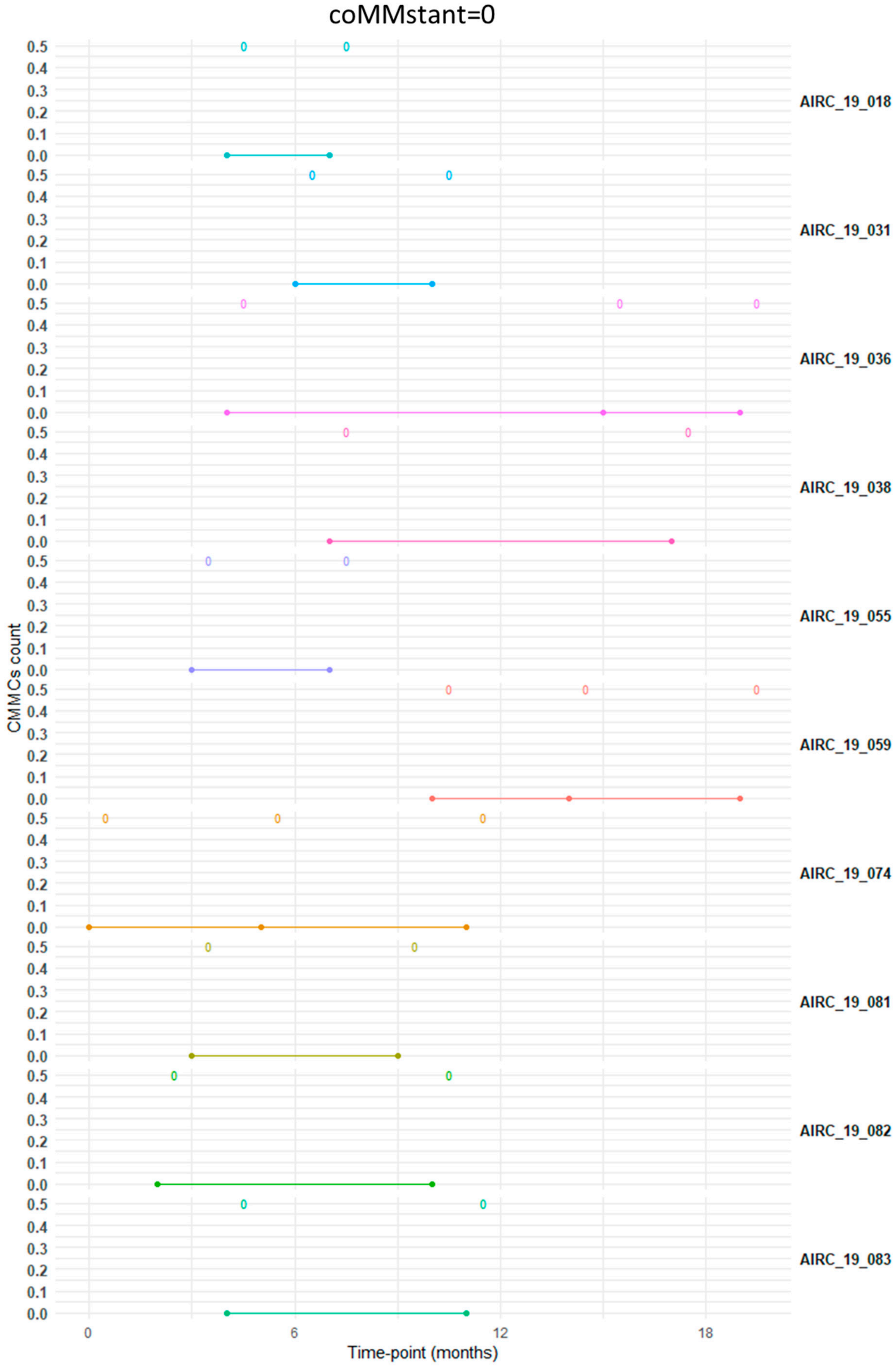


**Figure S3.** Line plot of CMMCs kinetics over time (in months) highlighted *per* patient in the context of coMMstant index=0.

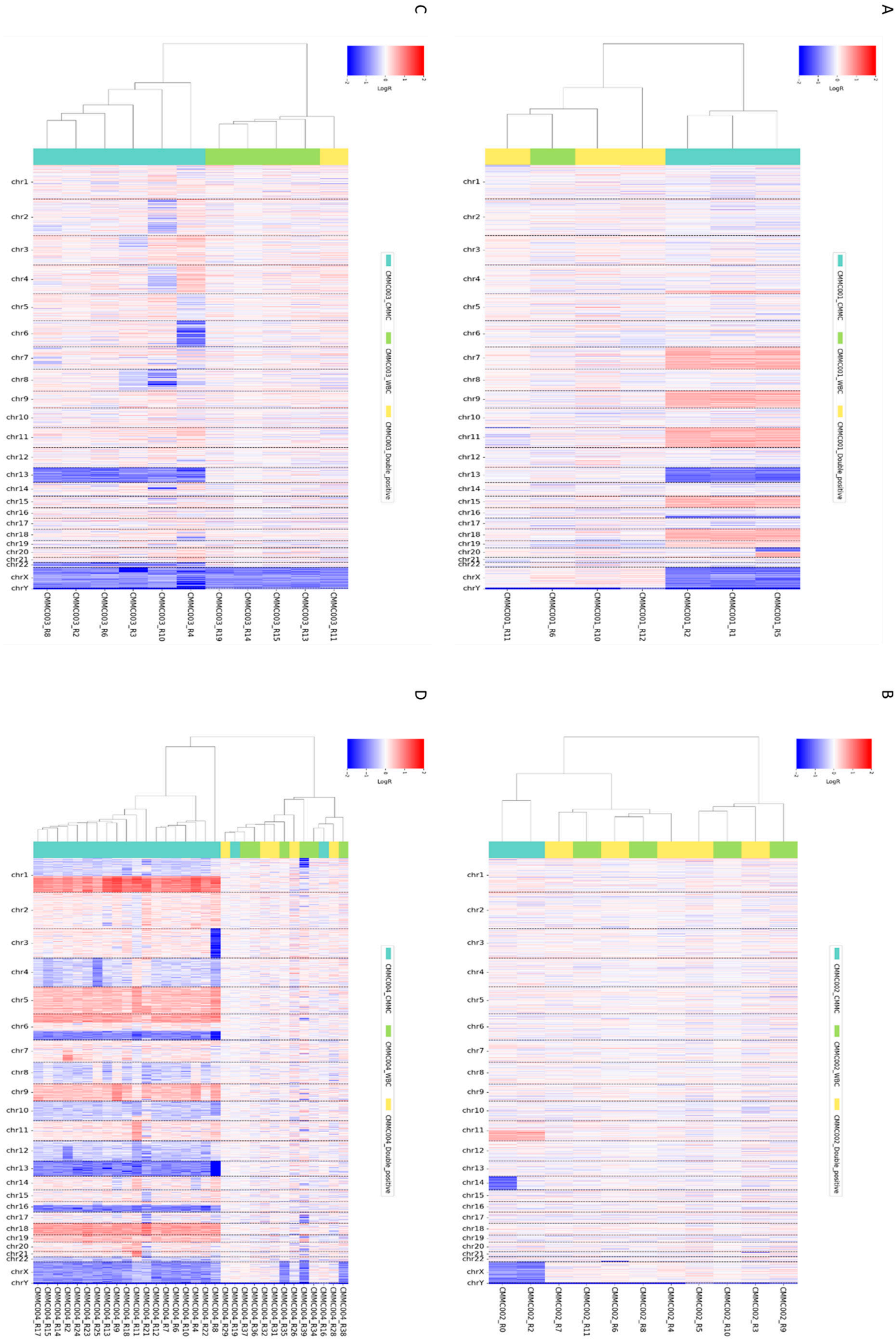


**Figure S4.** Heat-maps panel of CMMCs single-cell genomic analyses. A) Patient AIRC19_031 (CMMC1) CNAs profiles; B) AIRC19_24 (CMMC2); C) Patient AIRC19_28 (CMMC3) heat-map graph; D) Patient AIRC19_20 (i.e., CMMC4) CN variations profile. In blue deletions are displayed, while in red amplifications for each chromosome are analyzed.

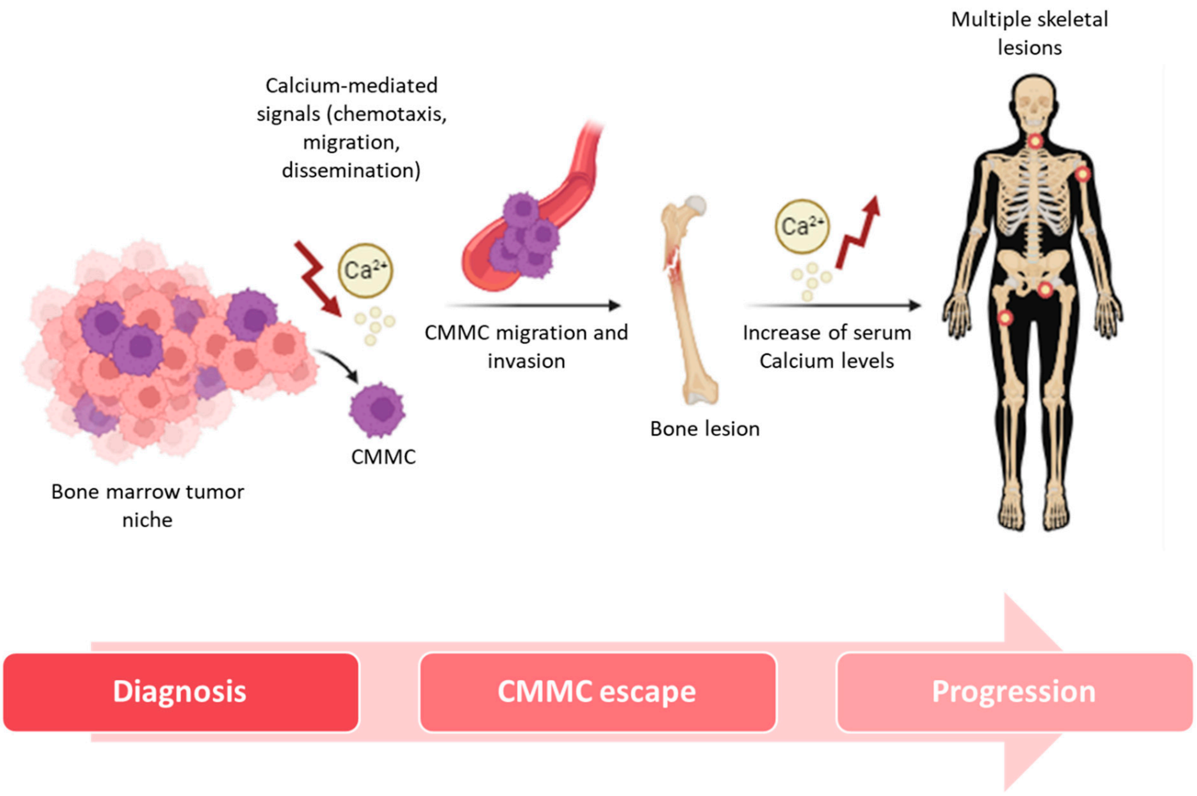


**Figure S5.** Schematic design of the CMMCs released from the BM niche, subsequent to a Calcium-induced pathway promoting chemotaxis and migration. CMMCs then colonize and induce bone lesions causing a subsequent increase in Calcium levels. Design was done by BioRender® (https://app.biorender.com/).
